# Increased regulatory T cells in peripheral blood of children with eosinophilic esophagitis

**Published:** 2021

**Authors:** Mahnaz Abdolahi, Shima Rasouli, Delara Babaie, Naghi Dara, Farid Imanzadeh, Aliakbar Sayyari, Pejman Rouhani, Katayoun Khatami, Maryam Kazemiaghdam, Yalda Nilipour, Maliheh Khoddami, Farah Ghadimi, Fatemeh Mousavinasab, Mehrnaz Mesdaghi

**Affiliations:** 1 *Pediatric Pathology Research Center, Research Institute for Children’s Health, Shahid Beheshti University of Medical Sciences, Tehran, Iran. *; 2 *Department of Immunology, School of Medicine, Shahid Beheshti University of Medical Sciences, Tehran, Iran. *; 3 *Department of Allergy and Clinical Immunology, Mofid Children’s Hospital, Shahid Beheshti University of Medical Sciences, Tehran, Iran. *; 4 *Pediatric Gastroenterology, Hepatology and Nutrition Research Center, Research Institute for Children’s Health, Shahid Beheshti University of Medical Sciences, Tehran, Iran*

**Keywords:** Regulatory T cells, Eosinophilic esophagitis, Gastroesophageal reflux disease, Peripheral blood mononuclear cells

## Abstract

**Aim:**

Considering the allergic basis of Eosinophilic esophagitis (EoE), this study was conducted to evaluate peripheral blood Tregs in children with EoE.

**Background:**

Eosinophilic esophagitis (EoE) is an allergic inflammatory disease of gastrointestinal tract. Regulatory T cells (Tregs) have a confirmed role in allergic disorders.

**Methods:**

Children with EoE, gastroesophageal reflux disease (GERD) and healthy controls (HC) (10 subjects in each group) were recruited after diagnosis by a pediatric gastroenterologist and allergist. After obtaining informed written consent, peripheral blood was obtained. Peripheral blood mononuclear cells were isolated by Ficoll gradient centrifugation. Flowcytometry was used to enumerate peripheral blood Tregs (CD_4_^+^CD_25_^+^FOXP3^+^ gated lymphocytes were considered as Tregs).

**Results:**

CD4^+^ gated lymphocytes significantly increased in EoE and GERD groups compared to HC group (*p*= 0.018). Tregs also was significantly increased in EoE in comparison to HC group (*p*=0.016). There were no statistically significant differences in Tregs of EoE as compared to GERD subjects (*p*=0.085).

**Conclusion:**

Peripheral blood Tregs increase in patients with EoE as compared to healthy controls, which may be indicative of a feedback mechanism to regulate inflammatory responses.

## Introduction

 Eosinophilic esophagitis (EoE) is one of the subtypes of eosinophilic gastrointestinal disorders (EGID) caused by food allergy with eosinophilia of the digestive tract presenting in affected subjects with abdominal pain, diarrhea, vomiting, and dysphagia (1). With an ever-increasing rise in allergies, EoE can be regarded as a major global health concern since the prevalence of EoE was reported in a 15-year study in Denmark with a 20-fold increase from 0.13 to 2.6 per 100,000 population (2).The hallmarks of EoE include augmented eosinophils in the mucosal layer of the esophagus, widespread epithelial hyperplasia, and a history of atopic diseases. Abovementioned characteristics distinguish EoE from other esophageal disorders, especially GERD as a non-allergic disease with clinical symptoms similar to EoE in which inflammatory involvement of esophagus results from acid reflux (3, 4). Moreover, despite the confirmed evidence of food and respiratory allergies determined by the Skin-Prick test and allergen-specific IgE measurements, the etiology of the disease is still not fully understood (5, 6).

Numerous studies have shown that Treg cells as main producers of Transforming Growth Factor beta 1 (TGFβ1) and Interleukin 10 (IL-10) play an important role in protecting against allergic diseases, thus the alteration in Treg cells is associated with eosinophilic allergy (4, 7, 8). In a previous study, significant increase in the FOXP3 expression on esophageal tissue and the number of Treg cells was shown not to be related to larger peripheral blood Treg population in subjects with EoE as compared to those with GERD and healthy controls (7). However, Tantibhaedhyangkul *et al.* revealed a significant rise in the Treg cell population in the esophageal tissue of both EoE and GERD patients as compared to healthy subjects (8). In addition, a significant increase in the Tregs of esophageal biopsy of patients with EoE was detected as compared to healthy controls (9). Moreover, we also showed a significant increase in the serum levels of TGFβ1 in EGID patients and in the serum levels of IL-10 in EGID patients with upper gastrointestinal eosinophilic involvement as compared to patients with GERD and healthy control groups (10).

Conflicts in the results of previous studies encouraged us to investigate CD_4_^+^/CD_25_^+^/FOXP3^+^ lymphocytes in children with EoE as compared to age- and sex-matched children with GERD and healthy controls. 

## Methods

**Study population and ethical considerations**

This study was approved by the ethics committee of Shahid Beheshti University of Medical Sciences. Written informed consent was obtained from the parents of thirty children (10 with EoE, 10 with GERD, and 10 healthy control subjects) included in the study. All included subjects were referred to the Allergy Clinic for evaluation by an allergist expert for food allergic disorders. Diagnosis were based on clinical symptoms, endoscopic findings, and biopsy confirmation indicative of elevated infiltrated eosinophils in lamina propria (11).

The inclusion criteria were as follows: poor feeding, dysphagia, vomiting and food impaction; a history of atopy in the patient or their family; a documented history of the pathology report indicative of an increase in eosinophils (at least 15 eosinophils/hpf); thicker basal layer; and elongation of vascular papilloma in obtained esophageal tissue. 

GERD was confirmed in children having the conditions below:

- Irritability, heartburn and chest pain
- Being responsive to proton pump inhibitors (PPIs) treatment
- Esophageal endoscopic biopsy containing <15 eosinophils per high power field (hpf)

Age- and sex-matched healthy children referring to the hospital for routine check-up without any symptoms and history of allergy were selected as the healthy control (HC) group. Participants with a history of autoimmune disorders, use of immunosuppressives, steroids and NSAIDs were excluded from the study. The age and sex of the patients and the number of eosinophils/ HPF in biopsies was recorded from the pathologic results of the patients.

**Collection of the samples, isolation and preparation of peripheral blood mononuclear cells (PBMCs)**

Approximately 5 ml of venous blood were taken manually using heparinized syringes and processed within 4 hours. Peripheral blood mononuclear cells (PBMCs) were isolated using density‐gradient centrifugation over Ficoll‐Histopaque 1077 (Sigma Aldrich; St. Louis, MO). Briefly, the blood was mixed with incomplete RPMI medium (1:1) and carefully poured into a tube containing Ficoll 1077. The tubes were centrifuged, and PMBCs were isolated and washed.

**Flowcytometry**

PBMCs (10^6^ cells) were incubated with FITC mouse Anti human CD_4_, PE mouse Anti human FOXP3, and PE-cy5 mouse Anti human CD_25_ (all from BD Biosciences) according to the manufacturer's instructions. After staining, the detection was performed using FACS Analyzer (BD FACSCalibur^TM^ flow cytometer, USA). Flow Cytometry analysis was performed via Flowjo 7.6.1 software.

**Statistical analyses**

Statistical Package for the Social Sciences, SPSS Version 22.0 software (SPSS Inc., Chicago, USA), was used for statistical analyses. ANOVA procedure was run to compare mean values ​​with normal distribution, and Kruskal-Wallis was used to compare variables with non-normal distribution. In this study, the confidence intervals of 95% and *P <0.05* were considered statistically significant.

## Results

A total of thirty patients (n=10 in each group) were studied. The mean±SD of age in subjects with EoE, GERD and HC were 4.1±3.03, 4.3±2.9, 4.2±3.32 years, respectively, which were not statistically different (*p*=0.990). Male to female ratio was not statistically different (*p*=0.621) in EoE, GERD, and HC groups (8:2, 6:4, and 7:3, respectively). 

**Figure 1:**
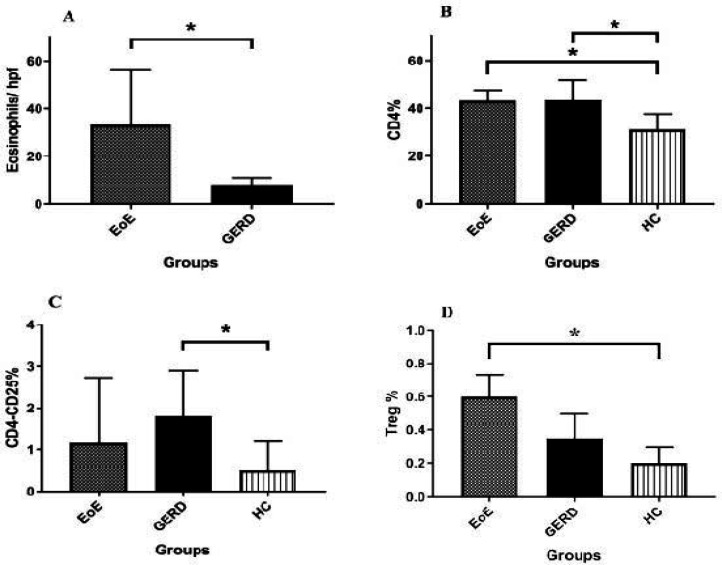
CD_4_^+^, CD_4_^+^CD_25_^+^ and CD_4_^+^CD_25_^+^FOXP3^+^ gated lymphocyte and eosinophil counts were compared using Kruskal Wallis Test; A, Eosinophils / HPF in esophageal biopsy of patients with EoE and GERD (p=0.0001). B, CD_4_^+^ gated lymphocytes of EoE, GERD and HC groups (p= 0.018). C, CD_4_^+^CD_25_^+^ gated lymphocytes of EoE, GERD and HC groups (p= 0.079), D, CD_4_^+^CD_25_^+^FOXP3^+^ gated lymphocytes of EoE, GERD and HC groups (p= 0.016). *:* P <0.05*

The number of eosinophils was confirmed to be significantly higher in EoE group (*p*=0.0001) (Fig.1A). The percentage of CD_4_^+^ gated lymphocytes was shown to be significantly higher in EoE and GERD subjects as compared to HC group (*p*= 0.018) (Fig.1B), and the percentage of CD_4_^+^CD_25_^+^ gated lymphocytes was significantly increased in GERD subjects in comparison to HC group (*p*=0.037) (Fig.1C). The CD_4_^+^CD_25_^+^ FOXP3^+^ gated lymphocytes (Tregs) varied in the range of 0.2 -0.8%, 0.1- 0.6% and 0.1 - 0.5% in EoE, GERD and HC groups, respectively. There were no significant differences in Tregs of EoE and GERD pediatrics (p=0.085), but patients with EoE had significantly more Tregs comparing HCs (*p*= 0.016) (Fig.1D).

## Discussion

EoE is a chronic allergic disorder of esophagus associated with eosinophil rich inflammation. Studies have been conducted on the regulation of immune responses in EoE. Among the investigated cells and molecules, Treg cells are of great interest. This attention comes from the proven key role of Tregs in controlling food allergies with reported reduction of tolerance to food antigens. Given that the pathophysiology of EoE remains unclear and the differential diagnosis between EOE and GERD is still challenging due to the overlap of these two diseases, in this study, blood CD_4_^+^, CD_25_^+^, FOXP3^+^ gated lymphocytes (Tregs) were studied in two groups of age and sex matched children with GERD and EoE as compared to healthy controls.

Previous studies have indicated that EoE and GERD symptoms appear in childhood, which is consistent with the reported mean (±SD) age of patients. In addition to age, gender is another known disease-related factor since it has been shown that EoE is more common in men (approximately 75% of patients) (12). In this regard, in a recent genome wide association study, the association of the Xq28 locus with EoE was demonstrated using High-depth RNA sequencing (13). Similarly, 80% of EoE subjects in the current study were male and this ratio was lower in patients with GERD. The recent finding is also in concordance with previous studies implying the predominance of the disorder in males through an unknown mechanism. Some studies have attributed the higher prevalence of the disease in men to higher levels of steroid hormones in women which helps inactivation of the inflammatory cells (14).

The recruitment of eosinophils, as key players in the pathogenesis of EoE, to esophagus is induced by the overexpression of eotaxin-3 (the main connector of Th2 activity and inflammation) (15). Activation and degranulation of these eosinophils as a major histopathologic finding of EoE results in the release of immunomodulatory factors. Among them, accumulation of inflammatory agents may result in the differentiation of Treg cells at the site of inflammation to achieve homeostasis. Moreover, it has been recently proven that eosinophils may aid differentiation of Treg cells through the production of Transforming Growth Factor -beta1 (TGF-β1) and all-trans retinoic acid (ATRA) (16).

In this study, the number of Tregs significantly increased in patients with EoE in comparison to healthy controls. Although EoE group had slightly more Tregs than GERD group, this difference was not significant (Fig 1). Our previous report, using immunohistochemistry (IHC) study of esophageal biopsy has shown a significant increase in the population of the T cells and Treg cells in children with EoE in comparison to the GERD and HC groups (both P <0.001) (9). This difference results from the difference in obtained samples since biopsy from affected tissue could be a more reliable source and also the increase in Tregs in the affected tissue is not systemic. Taken together, it seems that the number of Tregs rise in EoE as confirmed in esophageal biopsy and potentially in the peripheral blood. In addition, a previous study by Babaei and colleagues at the same center validated recent findings based on larger Tregs in EoE (10). They showed elevation in serum levels of TGF-β1 (*p*=0.025) in subjects with EGID and also a significant increase in serum levels of IL-10 in EGID with upper GI involvement (EoE and eosinophilic gastro-duodenitis) as compared to those with GERD and HC groups. Moreover, a reason for the increase of Tregs is the homeostatic feedback to inflammatory factors such as IL-17. In this regard, Babaei *et al. *revealed a significant increase in serum IL-17 in EGID with upper GI involvement including EoE pediatrics as compared to those with GERD and HC groups (17).

Previously, other studies demonstrated elevated Tregs in the esophageal biopsy of children with EoE (7, 8). Surprisingly, there is evidence of reduced Tregs in esophageal biopsies of adults with EoE in comparison to HC group (18). This could be due to alternative pathophysiology of EoE in adults compared to children. Notable, there are controversial results from other studies as compared to the present and our previous study (9). For instance, Fuentebella *et al.* showed significant increase of esophageal eosinophils, Tregs and CD_3_^+^ cells in EoE patients compared to those with GERD (p<0.05 for all) using IHC, but the flowcytometric analyses of peripheral blood did not reveal any statistically significant increase in circulatory Tregs for EoE subjects (p=0.60) (7). Finally, larger CD_4_^+^ gated lymphocyte population in Children with EoE could be justified by the role of the TSLP induced Th2 polarization similar to other atopic diseases (19). However, there is no justification for the increase in the number of CD_4_^+^, CD_25_^+^ gated lymphocytes in those with GERD and future studies can investigate the issue.

To conclude, differentiating EoE from GERD through the evaluation of circulatory Tregs is not feasible, and further studies are needed to find biomarkers for differential diagnosis. All these data confirm the larger number of Tregs in EoE, and future studies should focus on the functional and suppressor activity of Tregs isolated from blood and esophageal tissue of subjects with EGID for a better understanding of the disease mechanism.
